# Simultaneous Occurrence of Colonic Mucosa-Associated Lymphoid Tissue (MALT) Lymphoma (MALToma) and Lung Cancer

**DOI:** 10.1155/2018/3607101

**Published:** 2018-12-09

**Authors:** Monjur Ahmed

**Affiliations:** Thomas Jefferson University, Philadelphia, PA, USA

## Abstract

Colonic MALToma accounts for 2.5% of all MALTomas. MALToma can be associated with certain chronic infections, autoimmune disorders, Waldenstrom's macroglobulinemia, and old age. Synchronous MALTomas can occur in multiple organs. Simultaneous occurrence of colonic MALToma and colon cancer has been reported. A case of colonic MALToma and lung cancer is described here.

## 1. Introduction

Colonic MALToma is rare in our clinical practice. Although colonic MALToma can be completely asymptomatic, patients may present with different symptoms when the size of MALToma increases. Many cases of colonic MALToma are diagnosed as an incidental finding. Associated malignancy like lung cancer has never been reported.

## 2. Case

An 83-year-old white male presented with melena 3 to 4 times per day and occasional hematochezia over one week. He was known to have atrial fibrillation for which he was on warfarin at home. His INR was supratherapeutic (4.9) few weeks ago at his primary care physician's office. He was told to hold his warfarin for 2 days and then restart at a lower dose.

Recheck of INR was 2.9 after few days. But he started having melena. His other medical problems included significant cardiac history of coronary artery disease, status post coronary artery bypass graft x 3 in 1980, coronary angioplasty and stent placement in 2004, hypertension, hyperlipidemia, and prostate cancer, status post prostatectomy. He was a social drinker and a smoker. He was active in his daily life. His family history was not significant. His home medications were warfarin, sotalol, valsartan, atorvastatin, ezetimibe, isosorbide mononitrate, folic acid, cholecalciferol, ascorbic acid, and selenium.

**Figure 1:**
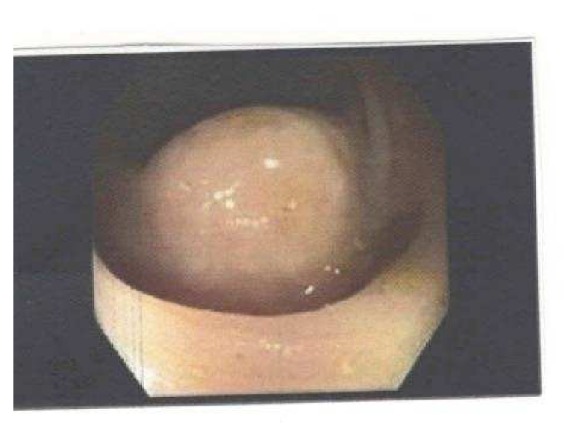
Nodular area in transverse colon.

Examination showed pulse 62/minute, blood pressure 118/67 mm Hg, pale conjunctiva, and abdomen that was soft, nontender, and with no palpable mass. Rectal examination revealed melanotic occult blood positive stool. The remainder of the examination was unremarkable. Laboratory studies showed hemoglobin 7 gm/dl, white cell count 11,400/cmm, and platelet count 156,000/cmm. Patient was started on intravenous pantoprazole. EGD showed mild bulbar duodenitis and a small superficial ulcer and multiple nonbleeding angioectasias in the gastric antrum. The angioectasias were ablated by argon plasma coagulation. Antral biopsy was negative for* H. pylori* infection. Colonoscopy showed moderate sigmoid diverticulosis, 1.5 cm pedunculated sigmoid colon polyp which was snared and retrieved. There was a subcentimeter nodular area with abnormal vascular pattern in the transverse colon (Figure 1).

**Figure 2:**
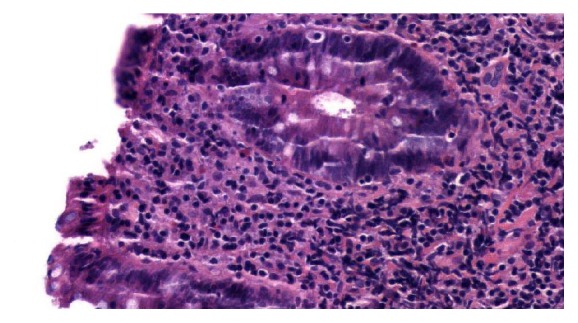
Marked small lymphocytic infiltrate obscuring normal colon architecture.

Polyp histology came back as tubular adenoma and the nodular area showed marked small lymphocytic infiltrate predominantly comprised of B-cells with lymphoepithelial lesion (Figure 2). The B cells were negative for CD5, CD10, BCL-1, and BCL-6. CD 21 highlighted expanded and disrupted follicular dendritic meshwork. Concurrent molecular studies detected a clonal immunoglobulin heavy chain (IGH) rearrangement. These findings were consistent with extranodal marginal zone lymphoma or colonic MALToma.

**Figure 3:**
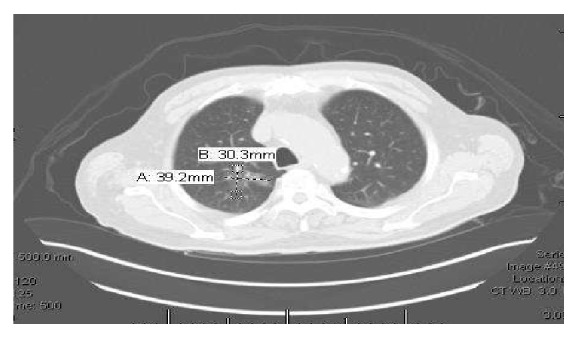
CT scan showing a 3.9 × 3 cm mass in upper lobe of right lung.

**Figure 4:**
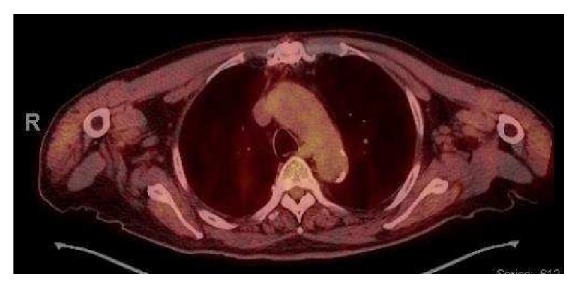
PET scan showing a right upper lobe lung mass without any hypermetabolic activity.

**Figure 5:**
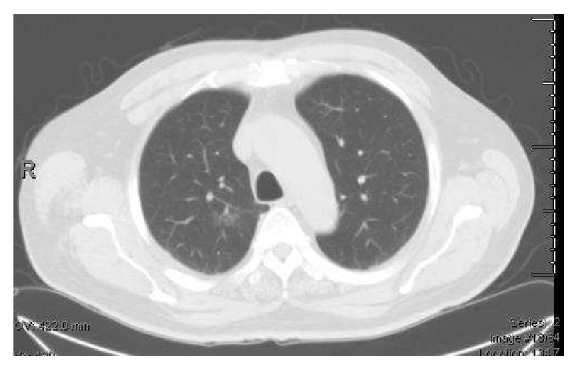
CT scan showing a 1.9 × 1.1 cm right upper lobe lung mass.

PET/CT scan showed a 3.9 × 3.0 cm ground glass opacity in the upper lobe of right lung (Figures 3 and 4), which had increased in size since the prior CT done 8 years ago when it was measured 1.9 × 1.1 cm (Figure 5). The lesion was suggestive of low-grade adenocarcinoma of the lung as it did not have any hypermetabolic activity. There was no focal hypermetabolic activity in the liver or abdomen. The patient refused to have any biopsy of the lung mass. He was seen by an oncologist for further management. He did not want to have any surgery or chemotherapy for his lung cancer. The patient was recommended to have another colonoscopy done in 6 months' time for follow-up of his colonic MALToma.

## 3. Discussion

MALToma also known as extranodal marginal zone lymphoma, accounts for 7 to 8% of all cases of B-cell lymphoma [1]. Gastric MALToma is the commonest site. Nongastric sites of MALToma include small intestine, colon, rectum, lung (bronchus-associated lymphoid tissue), salivary gland, thyroid gland, skin, and eye [2].

Patients may remain completely asymptomatic or may present with various symptoms like gastrointestinal bleeding, abdominal pain, diarrhea, fever, night sweats, anorexia, weight loss, and anemia [3]. Colonoscopically we may find a polypoid lesion, mass lesion, elevated lesion, ulcerative lesion, flat lesion, erythema with ecstatic blood vessels, and thickening of mucosal folds [4–6]. Histologically, as per the World Health Organization, MALToma is characterized by a lymphocytic infiltrate in the marginal zone of lymphoid follicle and interfollicular region. The infiltrate is composed of morphologically heterogeneous small B cells, which include marginal zone cells, small lymphocytes, cells resembling monocytes, scattered immunoblasts, and centroblast-like cells [7]. Another typical finding of MALToma is the lymphoepithelial lesion characterized by invasion and disruption of adjacent glandular epithelium by the neoplastic cells with morphological changes within epithelial cells [8].

The monoclonality of a particular MALToma can be confirmed by monoclonal IGH gene rearrangement in molecular study [9]. The treatment of colonic MALToma is not yet standardized. At the present time, surgery and chemotherapy are the main lines of treatment. Single agent chemotherapy with Rituximab was found to be successful in a case series [10]. An asymptomatic small sized colonic MALToma can be managed conservatively with periodic follow-up [3].

There are some predisposing factors for the development of MALToma. These include chronic infection like* H. pylori*-induced chronic gastritis (gastric MALToma),* Borrelia burgdorferi* infection (cutaneous MALToma),* Chlamydia psittaci* infection (lacrimal gland MALToma), autoimmune disorders like Sjogren's syndrome, rheumatoid arthritis, systemic lupus erythematosus, (Hashimoto's thyroiditis, relapsing polychondritis, and Wegener's granulomatosis [11, 12]. MALToma can also be associated with old age, weak immune system, and Waldenstrom's macroglobulinemia [13]. Synchronous MALToma of colon and adenocarcinoma of colon and synchronous MALToma of colon and stomach as well as synchronous MALToma of colon, stomach, and lung have been reported in the literature [14–16].

The lung mass seen in our case has differential diagnoses of lung cancer vs. MALToma. But lack of hypermetabolic activity on PET scan suggested lung cancer. The case we described is unique in finding the simultaneous occurrence of colonic MALToma and low-grade lung cancer (suspected) suggesting that another malignancy should be looked for if no predisposing factor for MALToma is obvious.
